# Sociodemographic Inequalities in Healthy Food Consumption Markers Among Older Adults Receiving Primary Health Care in Roraima, Brazil

**DOI:** 10.3390/geriatrics11050132

**Published:** 2026-09-15

**Authors:** Guilherme José Silva Ribeiro, Gabrielle Andrade da Silva, Nalva de Paula Dias, Rafaela Nogueira Gomes de Morais, Gabriela Rocha dos Santos, André de Araújo Pinto

**Affiliations:** 1 Department of Nutrition and Health, Federal University of Viçosa, Viçosa 36570-900, MG, Brazil; gabrielle.and.nutri@gmail.com (G.A.d.S.); nalvadepauladias@gmail.com (N.d.P.D.); rafaela.morais@ufv.br (R.N.G.d.M.); 2 Department of Psychiatry, Federal University of Rio Grande do Sul, Porto Alegre 90010-150, RS, Brazil; gabirochanutrirs@gmail.com; 3 Department of Physical Education, State University of Roraima, Boa Vista 69306-530, RR, Brazil; andre.pinto@uerr.edu.br

**Keywords:** older adults, sociodemographic factors, healthy diet, primary health care

## Abstract

**Background/Objectives**: Noncommunicable diseases represent a major public health challenge, particularly among older adults. In this context, a healthy diet plays a key role in the prevention and management of these conditions. However, evidence on the associations between sociodemographic characteristics and previous-day food consumption markers among older adults remains limited. This study aimed to evaluate the association between sociodemographic factors and healthy food consumption markers among older adults in Roraima, Brazil. **Methods**: This cross-sectional study included 1322 older adults previously diagnosed with COVID-19 in the state of Roraima, Brazil. Sociodemographic information was obtained from electronic health records within the statewide Primary Health Care network. Healthy food consumption was assessed using the Food Consumption Markers Form from Brazil’s Food and Nutrition Surveillance System (Sistema de Vigilância Alimentar e Nutricional [SISVAN]). Multivariable Poisson regression models were fitted to examine potential associations. **Results**: Male participants had a lower prevalence of consuming fresh fruit + beans and fresh fruit + vegetables. Older adults who self-identified as Brown (pardo) had a higher prevalence of consuming fresh fruit + vegetables than those who self-identified as Yellow. Participants residing in the state capital showed a higher prevalence of consuming fresh fruit + beans, fresh fruit + vegetables, beans + vegetables, and all three food groups combined. **Conclusions**: Sociodemographic factors were associated with both individual and combined consumption of fresh fruit, beans, and vegetables among older adults in Roraima, Brazil.

## 1. Introduction

Markers of healthy food consumption, including fresh fruit, beans, and vegetables, are important indicators of healthy diets and sustainable food systems [1,2]. Among older adults, the daily consumption of these foods has been consistently associated with a lower risk of cardiovascular disease [3], viral infections [4], mental health disorders [5], and frailty [6], as well as reduced all-cause mortality [7]. Accordingly, the Dietary Guidelines for the Brazilian Population recommend the daily consumption of fresh fruit, beans, and vegetables while encouraging dietary variety and the consumption of these foods in different combinations [8].

Among older adults, the consumption of fresh fruit, beans, and vegetables is shaped by the complex interplay of multiple determinants [9]. From a social epidemiology perspective [10], sociodemographic characteristics, including sex, age, educational attainment, income, skin color/race, and place of residence, play an important role in shaping food consumption patterns [11]. Previous studies involving Brazilian older adults have shown that female sex, advanced age (≥80 years), and higher educational attainment (≥8 years of schooling) are associated with a greater likelihood and higher prevalence of fruit and vegetable consumption [12,13]. In contrast, another Brazilian study found that female sex and higher educational attainment (≥12 years of schooling) were associated with lower consumption of unprocessed foods [14]. Although previous studies have examined sociodemographic factors associated with food consumption among older adults in Brazil, evidence from Roraima and the Northern Region remains scarce, particularly within the statewide Primary Health Care network. The present study extends previous research by examining food consumption among older adults receiving care within this network and exploring potential geographic disparities between residents of the state capital and those living in other non-metropolitan. Furthermore, a distinctive feature of this study is the assessment of both individual and combined consumption of fresh fruit, beans, and vegetables, providing a more comprehensive characterization of these food consumption markers based on foods consumed on the day preceding the interview.

Given the rapid aging of the Brazilian population [15] and the increased susceptibility of older adults to chronic diseases and other adverse health conditions [16], investigating sociodemographic disparities in previous-day food consumption markers is essential from a social epidemiology perspective. Therefore, this study aimed to examine the association between sociodemographic factors and the consumption of fresh fruit, beans, and vegetables in a large and diverse sample of Brazilian older adults aged 60 to 100 years.

## 2. Materials and Methods

This cross-sectional study was conducted using secondary data from a three-month follow-up investigation of older adults previously diagnosed with COVID-19 in the state of Roraima, Brazil. The follow-up was conducted through telephone contacts by Primary Health Care professionals and focused on the occurrence of cardiovascular manifestations and sequelae after COVID-19, including fatigue, lower-limb edema, dyspnea, palpitations, and chest tightness. The data were provided by the Department of Epidemiological Surveillance (Departamento de Vigilância Epidemiológica [DES]) of the Roraima State Health Department. The present study represents a specific component of this investigation and focuses on available healthy food consumption markers and their sociodemographic correlates. The database was accessed in November 2022 after institutional authorization and contained no personally identifiable information. The study was conducted in accordance with the Declaration of Helsinki and approved by the Human Research Ethics Committee of the State University of Roraima (protocol code 5385012, approval date: 3 May 2022).

Roraima is located in northern Brazil and has the smallest population among the country’s 26 states and the Federal District. According to official population estimates, the state has approximately 652,713 inhabitants, of whom 68.9% reside in the capital city, Boa Vista. Approximately 50,000 residents are aged 60 years or older. Additional demographic information is available from the Brazilian Institute of Geography and Statistics [17].

**Figure 1:**
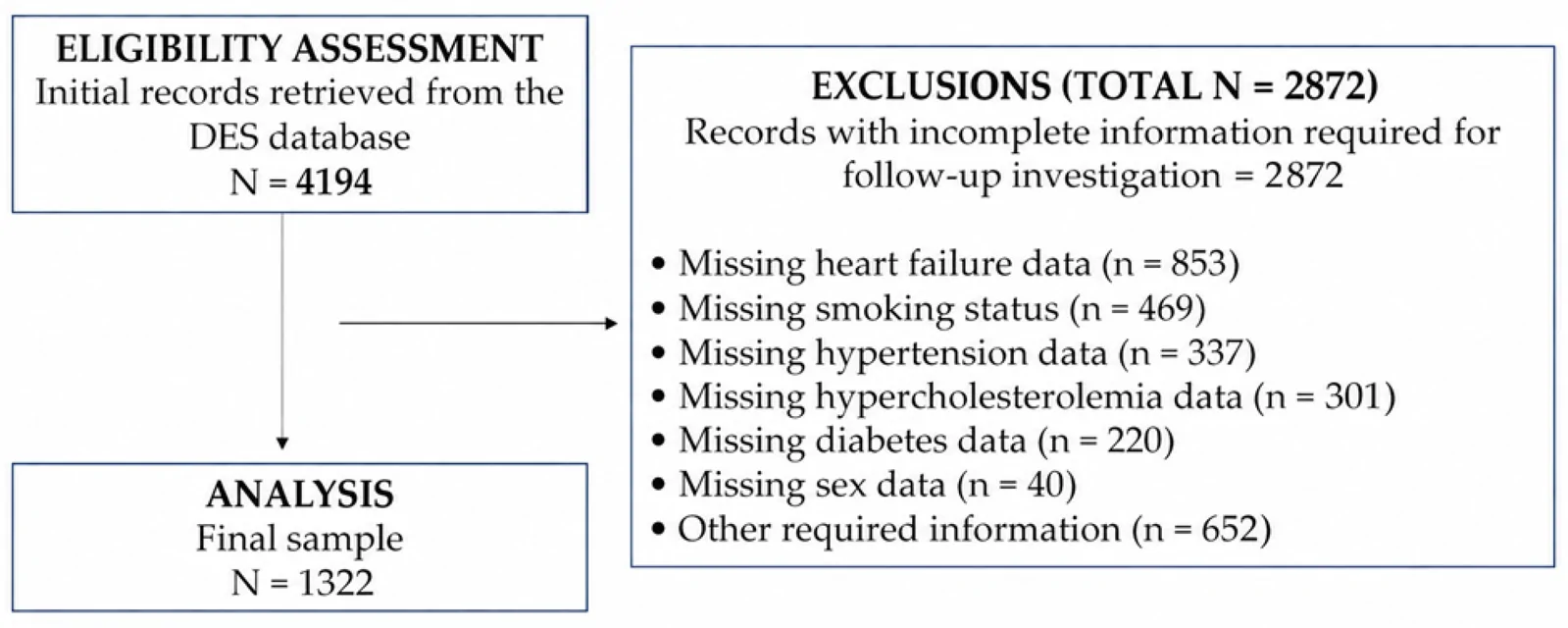
Participant flow diagram for selection in the DES database.

The study included individuals aged 60 years or older who were registered in the DES database and had participated in the Primary Health Care follow-up conducted during 2020. The database initially contained 4194 records from 108 of the 115 Primary Health Care centers operating in the state. The remaining seven centers were unable to participate because they lacked internet access, which was required to feed data into the DES system. During data screening, records with incomplete information required for the follow-up investigation were excluded. These exclusions comprised missing information on heart failure (n = 853), hypertension (n = 337), diabetes (n = 220), hypercholesterolemia (n = 301), smoking status (n = 469), sex (n = 40), and other required information (n = 652), resulting in 1322 complete records. The selection process is presented in Figure 1.

The minimum required sample size was calculated according to the methodological recommendations proposed by Luiz and Magnanini (2000) [18], assuming an expected prevalence of 50%, a 95% confidence level, a maximum acceptable margin of error of four percentage points, and a design effect (DEFF) of 2.0. An additional 20% was added to account for potential losses due to incomplete records. Based on these parameters, a minimum of 1260 complete records from older adults was required for the analytical sample. The final sample comprised 1322 older adults.

### 2.1. Study Variables

#### 2.1.1. Independent Variables: Sociodemographic Factors

The following sociodemographic characteristics were considered: sex (male or female); age, categorized into age groups (60–69, 70–79, and ≥80); skin color/race, classified according to the official Brazilian classification (Yellow, White, Brown (pardo), Black, and Indigenous); place of residence (the state capital or non-metropolitan); and educational level, categorized as (no study, up to 8 years, and more than 8 years). All sociodemographic variables recorded in the DES database were also included as adjustment variables in the multivariable analyses.

#### 2.1.2. Dependent Variables: Healthy Food Consumption Markers

Dietary intake was assessed using the Food Consumption Markers Form from Brazil’s Food and Nutrition Surveillance System (SISVAN), a nationally standardized instrument routinely used in primary health care to monitor dietary consumption markers among the Brazilian population for the previous day [19]. The questionnaire is a brief dietary screening tool developed based on the NOVA food classification system and aligned with the recommendations of the Dietary Guidelines for the Brazilian Population [8].

Although originally designed to support food and nutrition surveillance within health services, the instrument has been widely used in epidemiological research to assess dietary intake markers, including unprocessed, minimally processed, and ultra-processed foods [20,21]. Recent evidence has also demonstrated adequate psychometric properties, including a consistent two-factor structure and excellent construct validity indices (root mean square error of approximation [RMSEA] < 0.08; comparative fit index [CFI] and Tucker–Lewis index [TLI] > 0.95), supporting its application in population-based studies and the surveillance of dietary practices [1,22].

The study considered dietary intake markers based on the previous day’s consumption; each participant completed a single questionnaire covering the following food groups: beans, fresh fruit (excluding natural fruit juice), and vegetables (excluding potatoes, cassava, and yams). Data were collected by trained health professionals, typically nurses, using the standardized question: “Did you eat this yesterday?” Response options for each food marker were “yes,” or “no”.

In addition to analyzing each dietary marker individually, combined variables were created to represent the concurrent consumption of healthy foods. The combinations evaluated were fresh fruit and beans (fresh fruit + beans), fresh fruit and vegetables (fresh fruit + vegetables), beans and vegetables (beans + vegetables), and the simultaneous consumption of all three food groups (fresh fruit + beans + vegetables).

### 2.2. Statistical Analysis

All statistical analyses were performed using Stata version 17.0 (StataCorp LLC, College Station, TX, USA) [23]. Descriptive analyses were conducted to calculate absolute (n) and relative (%) frequencies. Multiple Poisson regression models with robust variance estimation were fitted to examine the associations between sociodemographic factors and the consumption of fresh fruit, beans, and vegetables, as well as combined food consumption markers, among older adults. Associations were expressed as prevalence ratios (PRs) with corresponding 95% confidence intervals (95% CIs).

## 3. Results

A total of 1322 older adults were included in this study. Most participants were female (55.0%), aged 60–69 years (53.9%), self-identified as Brown (pardo) (45.2%), had no formal education (47.2%), and resided in the state capital (64.1%) (Table 1).

**Figure 2:**
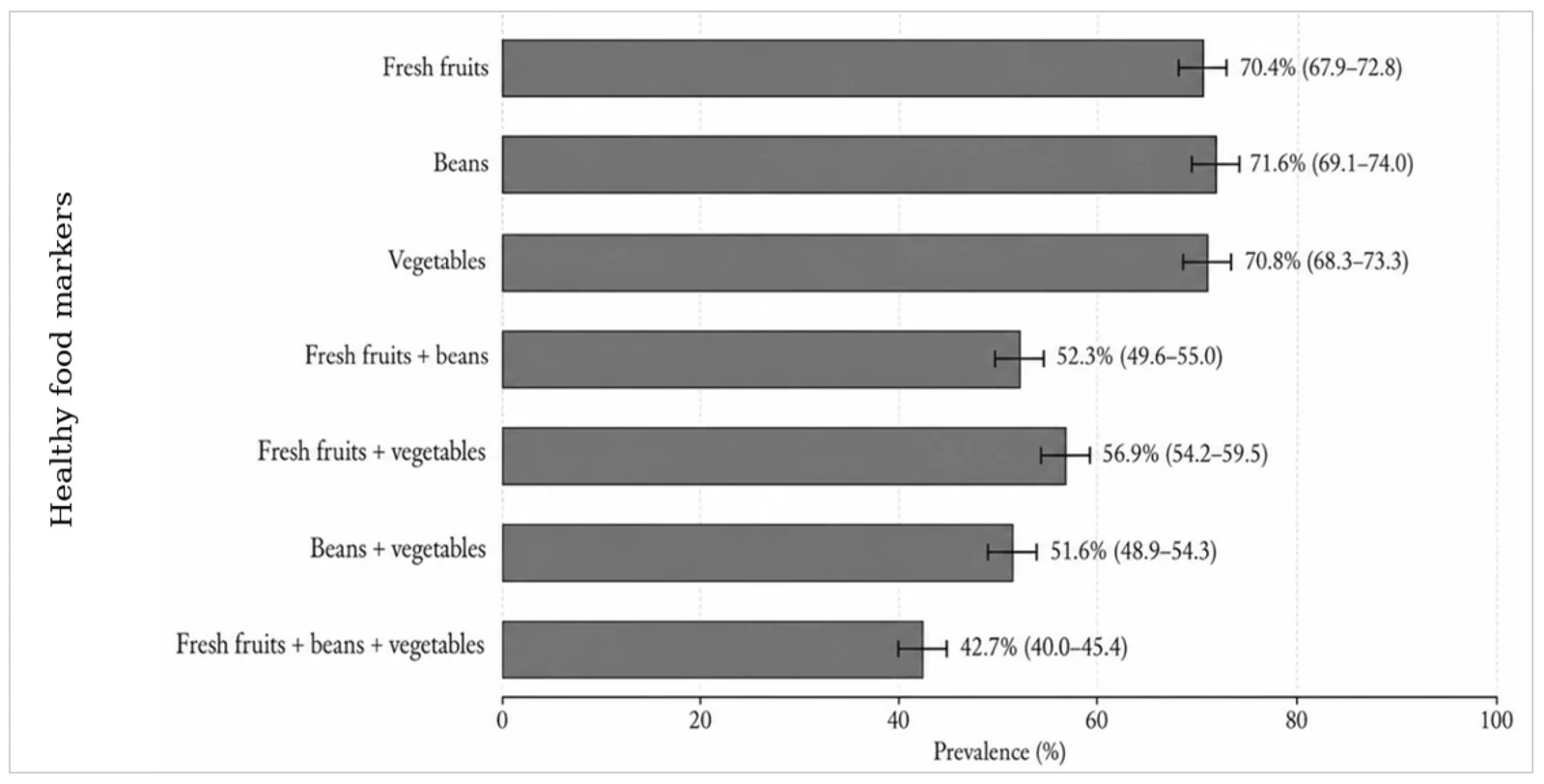
Percentage distributions and their respective 95% confidence intervals (95% CI) of individual and combined healthy food consumption markers among older adults in Roraima, Brazil, 2020.

Among the 1322 older adults, 70.4% reported consuming fresh fruit, 71.6% beans, and 70.8% vegetables on the previous day. Regarding the combined consumption of healthy food groups, 52.3% consumed fresh fruit + beans, 56.9% fresh fruit + vegetables, and 51.6% beans + vegetables. Fewer than half of the participants (42.7%) reported consuming all three food groups (fresh fruit + beans + vegetables) on the day preceding the interview (Figure 2).

The prevalence of healthy food consumption markers, assessed both individually and in combination, varied according to participants’ sociodemographic characteristics (Table 2).

### 3.1. Sex

Women had a higher prevalence of consuming fresh fruit (77.6% vs. 61.7%), vegetables (73.2% vs. 67.9%), fresh fruit and beans (56.1% vs. 47.6%), fresh fruit and vegetables (62.4% vs. 50.1%), and all three food groups combined (fresh fruit, beans, and vegetables) (45.0% vs. 39.8%) compared with men. Bean consumption was similar between sexes, with a slightly higher prevalence among men (72.4% vs. 71.0%), whereas the prevalence of consuming beans and vegetables was comparable between women (52.5%) and men (50.4%).

### 3.2. Age

Bean consumption increased progressively with advancing age, reaching 76.3% among adults aged 80 years or older. In contrast, the prevalence of fresh fruit, vegetables, and the combined dietary markers varied only slightly across age groups and remained relatively stable.

### 3.3. Skin Color/Race

Fresh fruit consumption was most prevalent among participants who self-identified as White (74.2%) and Brown (pardo) (73.4%). Bean consumption was highest among Black (77.0%) and Yellow (Asian) (73.8%) participants. The highest prevalence of vegetable consumption was observed among Brown (pardo) (76.4%) and White (76.2%) participants. Regarding the combined dietary markers, Brown (pardo) participants showed the highest prevalence of consuming fresh fruit + beans (56.3%), fresh fruit + vegetables (63.0%), beans + vegetables (58.0%), and all three food groups combined (49.4%). In contrast, Indigenous participants had the lowest prevalence of consuming fresh fruit + vegetables (38.6%), beans + vegetables (38.6%), and all three food groups combined (30.0%).

### 3.4. Educational Level

Participants with more than 8 years of schooling had the highest prevalence of consuming fresh fruit (78.5%), vegetables (80.5%), and fresh fruit and vegetables (68.3%). Conversely, bean consumption was most frequent among those with no formal education (76.8%).

### 3.5. Place of Residence

Participants residing in the state capital had a higher prevalence of consuming fresh fruit (74.4% vs. 63.4%), vegetables (74.0% vs. 66.0%), and all combined healthy food markers than those living in non-metropolitan. The largest difference was observed for the combined consumption of fresh fruit, beans, and vegetables (46.5% vs. 35.8%).

After adjustment, male participants had a lower prevalence of fresh fruit consumption than female participants (PR = 0.80; 95% CI: 0.74–0.86). Participants who self-identified as Indigenous had a lower prevalence of vegetable consumption than those who self-identified as Yellow (Asian) (PR = 0.73; 95% CI: 0.58–0.91). Regarding educational attainment, participants with more than 8 years of schooling had a higher prevalence of consuming fresh fruit (PR = 1.20; 95% CI: 1.09–1.32) and vegetables (PR = 1.14; 95% CI: 1.03–1.25). However, they showed a lower prevalence of bean consumption (PR = 0.85; 95% CI: 0.77–0.95). Older adults residing in the state capital had a higher prevalence of consuming fresh fruit (PR = 1.19; 95% CI: 1.10–1.29) and vegetables (PR = 1.14; 95% CI: 1.06–1.23) than those living in non-metropolitan (Table 3).

**Figure 3:**
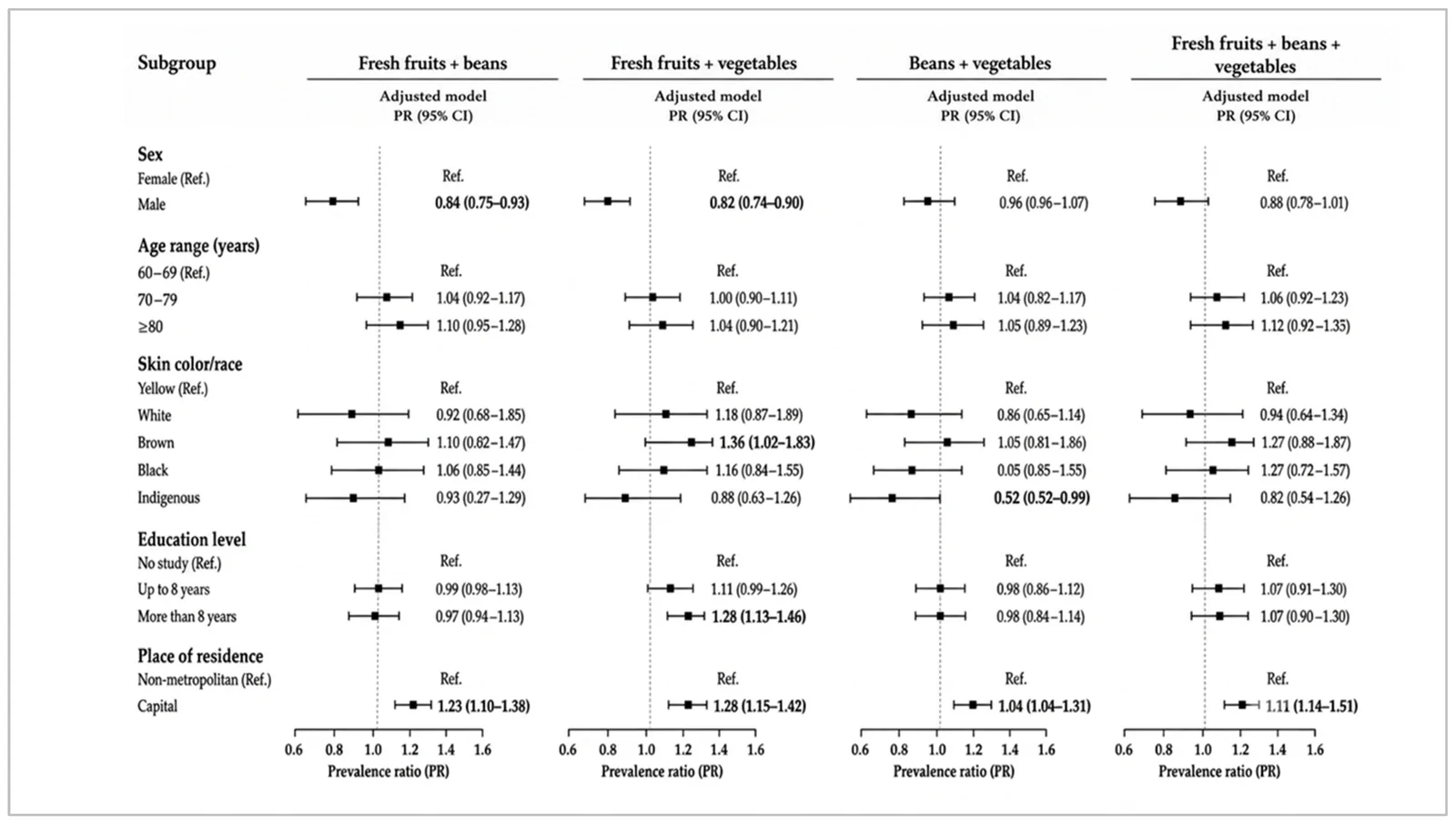
Adjusted Poisson regression models examining the associations between sociodemographic characteristics and combined healthy food consumption markers among older adults in Roraima, Brazil, 2020. PR, prevalence ratio; 95% CI, 95% confidence interval; Ref., reference category. Models were adjusted for sex, age, skin color/race, educational attainment, and place of residence. Statistically significant associations (*p* < 0.05) are shown in bold.

Regarding the combined healthy food consumption markers, after adjustment for potential confounders, male participants had a lower prevalence of consuming fresh fruit + beans (PR = 0.84; 95% CI: 0.75–0.93) and fresh fruit + vegetables (PR = 0.82; 95% CI: 0.74–0.90) than female participants. In addition, participants who self-identified as Brown (pardo) had a higher prevalence of consuming fresh fruit + vegetables than those who self-identified as Yellow (Asian) (PR = 1.36; 95% CI: 1.02–1.83). Participants residing in the state capital had a higher prevalence of consuming fresh fruit + beans (PR = 1.23; 95% CI: 1.10–1.38), fresh fruit + vegetables (PR = 1.28; 95% CI: 1.15–1.42), beans + vegetables (PR = 1.16; 95% CI: 1.04–1.31), and all three food groups combined (fresh fruit, beans, and vegetables) (PR = 1.31; 95% CI: 1.14–1.51) than those living in other non-metropolitan areas (Figure 3).

## 4. Discussion

In this cross-sectional study of a demographically diverse sample of Brazilian older adults aged 60 to 100 years, several sociodemographic characteristics were associated with markers of healthy food consumption, particularly sex, skin color/race, educational attainment, and place of residence.

Male sex was associated with a lower prevalence of fresh fruit consumption. These findings are consistent with previous studies conducted among older adults in Brazil [12,13], the United States [23], and Europe [24] and a multicountry study involving 28 low- and middle-income countries between 2005 and 2016 [25]. Our findings build upon previous evidence by examining older adults in Roraima, a region of Brazil currently underrepresented in dietary research. In the combined analyses, male participants also had a lower prevalence of consuming fresh fruit + beans and fresh fruit + vegetables. The observed sex differences may reflect distinct health-related behavioral patterns. Older men are more likely to adopt unhealthy lifestyle behaviors [26], be less concerned about diet quality [27], prepare meals at home less frequently [28], and make less frequent use of health care services [29]. Consequently, factors accumulated throughout the life course may influence dietary practices in older age. Dietary patterns established during earlier life stages may remain relatively stable in the face of important life-course transitions, such as retirement [30]. Furthermore, unfavorable economic conditions may be associated with diet quality among older adults, as lower socioeconomic status has been associated with poorer dietary quality in this population [31]. Other social and contextual factors, including living arrangements, widowhood, social support, and food insecurity, may also contribute to differences in dietary intake between older men and women [32,33]. In this context, the lower consumption of fresh fruits and vegetables may be related to a combination of experiences and conditions accumulated throughout the life course.

Indigenous skin color/race was associated with a lower prevalence of vegetable consumption and the combined consumption of beans + vegetables, whereas Brown (pardo) older adults showed a higher prevalence of combined consumption of fresh fruit + vegetables. These findings may reflect both socioeconomic inequalities and cultural aspects related to dietary practices [32]. National studies [13,33] and international research [34] indicate that racial disparities in food consumption are associated with socioeconomic inequalities, as the higher cost of healthy foods, such as fruits and vegetables, may favor the consumption of these foods among individuals with higher socioeconomic status. In contrast, other population groups may be more likely to consume foods associated with their cultural identities and shaped by conditions of access and availability. The relationship between skin color/race and dietary intake remains insufficiently explored in the literature, particularly when racial categories are analyzed in a disaggregated manner. Most studies classify participants into broad categories (e.g., White and non-White), which limits the identification of specific differences among groups such as Indigenous, Yellow (Asian ancestry), and Brown (pardo) populations. Therefore, further research is needed to investigate these associations in greater detail and improve understanding of dietary inequalities across different racial groups [35]. Furthermore, future studies should explore the potential roles of territorial displacement, remoteness, environmental pressures, and disruption of traditional food systems in shaping food consumption among Indigenous older adults, particularly considering that these factors were not directly measured in the present study.

Consistent with previous studies conducted in the Netherlands [36] and Brazil [14], having more than eight years of schooling was associated with a higher prevalence of fresh fruit and vegetable consumption, as well as with the combined consumption of these food groups. These findings may be explained by the fact that individuals with higher educational attainment tend to have greater food literacy, including the ability to plan, select, and prepare healthier foods, as well as a better understanding of the impact of diet on health [37]. Moreover, among adults aged 60 years or older, greater nutritional awareness and the use of nutrition information have been associated with better diet quality [27]. Conversely, the lower prevalence of bean consumption among individuals with higher educational attainment, also reported by [38], may reflect dietary transitions characterized by the replacement of traditional Brazilian foods, such as the consumption of rice and beans, with ultra-processed foods.

Older adults residing in the state capital had a higher prevalence of consuming fresh fruit and vegetables, as well as a higher prevalence of consumption of all three healthy food markers on the previous day (fresh fruit + beans + vegetables). This finding may be explained by the influence of the food environment, as greater availability and proximity to food outlets that sell healthy foods may facilitate healthier dietary choices [39]. Evidence from Brazilian older adults indicates that rural residents differ from urban residents in food consumption and food environment characteristics, including the availability of fruits and vegetables in their neighborhoods [40]. In addition, greater distance from food establishments selling predominantly fresh or minimally processed foods has been associated with lower regular consumption of fruits and vegetables among older adults [41]. These findings may be particularly relevant in Roraima, where territorial characteristics and geographic distances may create additional challenges for older adults living in more remote areas to access a diverse range of healthy foods. Such territorial conditions may also interact with social factors, including income and living arrangements, potentially influencing the ability to access and afford healthy foods. Nevertheless, these factors were not directly assessed in the present study and should be investigated in future research. National evidence also indicates regional differences in healthy eating markers among older Brazilian adults, with lower consumption of beans, fruits and vegetables observed in the North compared with the Southeast [42]. Therefore, the food environment represents an important determinant of dietary behavior and may influence the risk of developing diet-related chronic diseases [39].

From a public health perspective, these findings highlight the importance of addressing the social and economic determinants of healthy eating among the elderly in Roraima. Health managers and policymakers should strengthen nutrition-related initiatives within primary health care by identifying socially vulnerable older adults and facilitating their inclusion in food security and social protection programs. Strategies that include personalized dietary guidance, the promotion of locally available and affordable foods, and improved coordination between health and social services can help reduce inequalities in access to adequate nutrition and promote healthy aging.

This study has several strengths. Given that few investigations have examined the association between socioeconomic characteristics and specific healthy food consumption markers, our findings contribute to addressing an important gap in the literature. Furthermore, we used a validated instrument that is widely implemented within Brazil’s National Health System to assess participants’ dietary intake. Another strength is the combined assessment of these dietary markers, which provides a more comprehensive representation of participants’ previous-day food consumption. We also highlight the inclusion of an understudied region of Brazil.

Regarding limitations, the cross-sectional design prevents the establishment of causal relationships. Although validated questionnaires were used, self-reported information may be subject to recall bias. In addition, the SISVAN food consumption questionnaire does not allow for the assessment of the frequency or quantity of food intake. The lack of information on relevant social determinants, such as household income, food insecurity, marital status, and living arrangements, may have resulted in residual confounding. Furthermore, potential clustering of participants within Primary Health Care centers was not accounted for in the regression models, which may have led to underestimated standard errors. Finally, data were collected during 2020, amid the COVID-19 pandemic, which disrupted healthcare services and may have affected the completeness and availability of records, as well as food consumption patterns. Because information on pandemic-related circumstances was not available in the database, their potential influence on the findings could not be assessed.

## 5. Conclusions

Sociodemographic characteristics were associated with indicators of healthy food consumption among older adults, suggesting disparities in dietary habits across different population groups. These findings highlight persistent inequalities in reported healthy food consumption, underscoring the need for health interventions, including the promotion of adequate and healthy nutrition, tailored to specific population groups. Within the context of Primary Health Care, these results can inform targeted strategies to address dietary inequalities, while coordination with intersectoral public policies can help ensure equitable access to healthy foods.

## Figures and Tables

**Table 1:** Sociodemographic characteristics of older adults in Roraima, Brazil, 2020.

Variables	n	%
Sex		
Female	727	55.0
Male	595	45.0
Age range (years)		
60–69	712	53.9
70–79	416	31.5
≥80	194	14.7
Skin color/race		
Yellow (Asian)	61	4.6
White	302	22.8
Brown	597	45.2
Black	222	16.8
Indigenous	140	10.6
Education level		
No study	624	47.2
Up to 8 years	395	29.9
More than 8 years	303	22.9
Place of residence		
Non-metropolitan	475	35.9
Capital	847	64.1

**Table 2:** Prevalence of healthy food consumption markers according to sociodemographic characteristics among older adults in Roraima, Brazil, 2020.

Variables	Healthy Food Markers (%)						
	Fresh Fruit	Bean	Vegetables	Fresh Fruit + Bean	Fresh Fruit + Vegetables	Bean + Vegetables	Fresh Fruit + Bean + Vegetables
Sex							
Female	77.6	71.0	73.2	56.1	62.4	52.5	45.0
Male	61.7	72.4	67.9	47.6	50.1	50.4	39.8
Age range (years)							
60–69	72.2	70.1	72.7	51.5	59.3	51.0	42.3
70–79	67.8	72.1	68.9	51.9	54.1	52.2	42.5
≥80	69.6	76.3	67.5	55.8	54.1	52.6	44.3
Skin color/race							
Yellow (Asian)	60.7	73.8	70.5	47.5	44.3	52.5	36.1
White	74.2	63.6	76.2	48.0	61.6	48.3	39.1
Brown	73.4	73.0	76.4	56.3	63.0	58.0	49.4
Black	64.9	77.0	61.3	52.7	49.1	46.8	39.2
Indigenous	62.9	73.6	50.7	45.7	38.6	38.6	30.0
Education level							
No study	65.9	76.8	65.7	53.0	50.6	51.3	41.2
Up to 8 years	71.4	69.6	71.4	52.1	58.0	51.7	43.5
More than 8 years	78.5	63.7	80.5	50.8	68.3	52.1	44.5
Place of residence							
Non-metropolitan	63.4	71.4	66.0	45.7	48.6	46.7	35.8
Capital	74.4	71.8	74.0	56.0	61.5	54.3	46.5

**Table 3:** Association between sociodemographic characteristics and healthy food consumption markers among older adults in Roraima, Brazil, 2020.

	Healthy Food Markers					
Variables	Fresh Fruit		Bean		Vegetables	
	Crude Model PR (95% CI)	Adjusted Model PR (95% CI)	Crude Model PR (95% CI)	Adjusted Model PR (95% CI)	Crude Model PR (95% CI)	Adjusted Model PR (95% CI)
Sex						
Female	Ref.	Ref.	Ref.	Ref.	Ref.	Ref.
Male	**0.79 (0.74–0.86)**	**0.80 (0.74–0.86)**	1.02 (0.95–1.09)	1.00 (0.93–1.07)	**0.93 (0.86–0.99)**	0.95 (0.88–1.02)
Age range (years)						
60–69	Ref.	Ref.	Ref.	Ref.	Ref.	Ref.
70–79	0.94 (0.87–1.02)	1.00 (0.92–1.09)	1.03 (0.95–1.11)	0.99 (0.92–1.08)	0.95 (0.88–1.02)	0.99 (0.92–1.08)
≥80	0.96 (0.87–1.07)	1.05 (0.95–1.18)	1.09 (0.99–1.19)	1.02 (0.93–1.13)	0.93 (0.83–1.03)	0.99 (0.88–1.12)
Skin color/race						
Yellow (Asian)	Ref.	Ref.	Ref.	Ref.	Ref.	Ref.
White	1.22 (0.99–1.51)	1.09 (0.88–1.36)	0.86 (0.72–1.02)	0.89 (0.75–1.07)	1.08 (0.91–1.28)	0.98 (0.82–1.18)
Brown	1.21 (0.98–1.49)	1.18 (0.95–1.46)	0.99 (0.84–1.16)	0.97 (0.82–1.15)	1.08 (0.91–1.28)	1.05 (0.89–1.25)
Black	1.07 (0.85–1.34)	1.11 (0.88–1.39)	1.04 (0.88–1.23)	0.99 (0.84–1.19)	0.87 (0.72–1.05)	0.88 (0.73–1.07)
Indigenous	1.04 (0.81–1.32)	1.05 (0.83–1.35)	0.99 (0.83–1.19)	0.97 (0.80–1.17)	**0.72 (0.57–0.91)**	**0.73 (0.58–0.91)**
Education level						
No study	Ref.	Ref.	Ref.	Ref.	Ref.	Ref.
Up to 8 years	1.08 (0.99–1.18)	1.08 (0.99–1.18)	**0.91 (0.84–0.98)**	0.92 (0.85–1.00)	1.09 (0.99–1.18)	1.03 (0.95–1.13)
More than 8 years	**1.19 (1.10–1.29)**	**1.20 (1.09–1.32)**	**0.83 (0.75–0.91)**	**0.85 (0.77–0.95)**	**1.22 (1.13–1.33)**	**1.14 (1.03–1.25)**
Place of residence						
Non-metropolitan	Ref.	Ref.	Ref.	Ref.	Ref.	Ref.
Capital	**1.17 (1.08–1.27**)	**1.19 (1.10–1.29)**	1.00 (0.94–1.08)	1.00 (0.93–1.07)	**1.14 (1.05–1.23)**	**1.14 (1.06–1.23)**

Note: PR, prevalence ratio; 95% CI, 95% confidence interval. Models were fitted using Poisson regression with robust variance and adjusted for sex, age, skin color/race, educational attainment, and place of residence. Statistically significant associations (*p* < 0.05) are shown in bold.

## Data Availability

All data used and/or analyzed during the current study are available from the corresponding authors upon reasonable request.

## Abbreviations

The following abbreviations are used in this manuscript:

IBGE	Instituto Brasileiro de Geografia e Estatística
DES	Departamento de Vigilância Epidemiológica
SISVAN	Sistema de Vigilância Alimentar e Nutricional
